# Type-II heterostructure of semiconducting CdS nanoparticle-ZnO nanoflake arrays for visible light dependent enhanced photocatalytic activity

**DOI:** 10.1038/s41598-025-88141-x

**Published:** 2025-05-02

**Authors:** Amit Kumar Bhunia

**Affiliations:** Department of Physics, Government General Degree College Gopiballavpur-II, Jhargram, 721517 India

**Keywords:** Nanoflake, Semiconductor, Heterostructure, Charge transfer, Photocatalytic effect, Environmental sciences, Health care, Nanoscience and technology, Physics

## Abstract

Type-II heterostructure semiconductors are very attractive for optoelectronics, environmental and energy-related applications. In this report, the heterostructure (Hs) semiconductor nanocrystalline CdS-ZnO was grown by a cost-effective chemical precipitation method and study of photocatalytic performance from the view of type-II semiconductor heterostructure. The array of nano flake (NF)-particle (NP) morphology of the Hs was observed from FESEM images. Different optical parameters like refractive index, optical conductivity, energy functions, and others were studied from UV-Vis spectroscopy. Tuning of the excitonic peaks (355 nm to 464 nm), band gap energy (3.78 eV to 2.8 eV), and Urbach energy (1 eV to 2.35 eV) have been observed from optical spectroscopy. The crystal phase matching in the Hs has been verified from the observed hexagonal wurtzite structure of both CdS NPs and ZnO NPs. The greater ultrafast life time of the CdS NP-ZnO NF Hs (59 nS) was found compare with pure CdS NPs (17 nS) and ZnO NPs (4.41nS). The observed room temperature current in the heterostrcture enhanced heavily compare with pure ZnO NPs at any voltage (-10 V to + 10 V). A photocatalytic degradation test showed that the highest efficiency (≈ 95%) degradation of MB within 28 min was obtained using type II CdS NP-ZnO NF heterostructure (Hs) semiconductors compare with pure ZnO NPs (75%) and pure CdS NPs (83.5%) under visible light irradiation. This highly efficient activity of the HS was induced by enhanced charge separation and interfacial charge transfer in nanocrystal heterostructure semiconductors.

**Supplementary Information:**

The online version contains supplementary material available at 10.1038/s41598-025-88141-x.

## Introduction

One of the biggest issues we are now dealing with is environmental degradation. The water contaminate is a serious issue and its solution is a challenging task for researchers^1–3^. The environmental application of various semiconductor materials including nanomaterials (1D/2D/3D) as photo-catalysts for the decomposition of hazardous organic compounds and water purification has been the focus of a great deal of research^4–6^. These semiconductors are also employed in the water splitting process to produce hydrogen^7,8^. Nanostructure semiconductors with suitable band gap is currently attractive materials for the water purification and hydrogen production due to its enormous surface properties, optical response, confined related properties and many more^9,10^. Semiconductor photocatalysts with the highest solar radiation collecting capacity are essential for removing organic contamination from water bodies. Metal-oxide semiconductors, such as ZnO, TiO_2_, and α-Fe_2_O_3_ have been thoroughly studied as practically relevant photocatalysts among the numerous photocatalytic semiconductor materials because of their strong photocatalytic activity and affordable synthetic approaches^11,12^. These single metal-oxide photocatalysts, however, exhibit large band-gap energies (both in the bulk and nanoscale regions), which is detrimental to the absorption and utilisation of solar energy (visible light). Also, single metal oxide is not efficient charge donor during photocatalytic effect under visible light irradiation. Only radiation with shorter wavelengths (below 385 nm) can be generate to create electron–hole pairs, though, because ZnO has a wide band gap (Eg = 3.37 eV at ambient temperature)^13^. Thus, ZnO activity is mostly limited to the UV spectrum, which makes up only 4% of the solar spectrum. For two key reasons, composite nanoscale semiconductor photocatalyst systems (heterostructure) have drawn a lot of attention in an effort to enhance photocatalytic activity^14^. Firstly, by linking small band-gap semiconductor photosensitizers, broad band-gap semiconductors can harness visible light in composite nanoscale semiconductor systems with varying energy levels^14,15^. Second, by lowering electron-hole pair recombination, charge injection from one semiconductor nanostructure into another can result in longer and more effective charge separation^16^. Charge injection property is more prominent in nanostructures because of the large surface to volume ratio, large surface charge density, highly optical responses and other confinement effects. In order to increase ZnO’s spectrum response to visible light, several techniques have been used, including surface modification and transition metal doping^17,18^. However, the quick recombination of photogenerated electron-hole pairs on ZnO particles invariably obstructs the charge carriers’ ability to diffuse outward.

Consequently, another important feature that limits the photocatalytic efficiency is the prohibition of photocatalyzed degradation at the semiconductor/liquid interface. Numerous techniques have been used to improve the visible light sensitivity and charge separation of ZnO-based photocatalysts in order to address the two aforementioned disadvantages of ZnO.

The addition of two semiconductors with distinct redox energy levels in their respective valence band and conduction band to the photosensitization systems creates an attraction that improves charge carrier lifetime and allows for more effective charge separation^19^. It has been demonstrated that one workable method of enhancing the visible light sensitivity of ZnO-based photocatalysts is to couple ZnO with narrow band semiconductors, such as CdTe, CdS, ZnSe, and PbS. Given that CdS and ZnO have comparable lattice structures, which may foster intimate interaction between the two semiconductors, CdS is thought to be the most appropriate visible sensitizer for ZnO among them^20–23^.

Therefore, efficient inter band charge transfer from CdS to ZnO is facilitated by the ZnO/CdS heterostructure. Developing nanoscale ZnO/CdS hybrid materials for photocatalysis has been the focus of numerous studies to date, and the findings demonstrate that CdS-sensitized ZnO photocatalysts exhibit improved photocatalytic properties, including ZnO–CdS hierarchical structure, ZnO–CdS composite films, and one-dimensional ZnO–CdS composite^21–23^. Particularly because of their special qualities, CdS NPs-ZnO NPs or nanostructure CdS-ZnO with different morphology have emerged as an appealing class of materials. Tak et al. fabricated CdS Nanoparticle-ZnO nanowire type-II heterostructure for photocatalytic activity^24^. Meanwhile, the solar photocatalyst of CdS nanoparticle (NP)-ZnO nanoflake (NF) heterojunction has not been reported.

In this work, a simple two-step solution-based technique is followed to grow a unique type-II heterostructure array comprising CdS nanoparticle (NP) and ZnO nanoflake (NF). The grown semiconducting type-II heterostructure is used as a photocatalyst to study visible light dependent photocatalytic activity towards MB dye. Also, separately, CdS NPs and ZnO NPs have been grown with the use of the chemical precipitation technique and used as photocatalyst for the photocatalytic activity under the same conditions.

This intriguing heterostructure array demonstrated enhanced absorption of visible light as well as photocatalytic activity because of their type-II band alignments, which effectively separated charges.

## Experimental and instrumentations

### Fabrication of CdS NPs, ZnO NPs and CdS NP-ZnO NF heterostructure

**Fig. 1:**
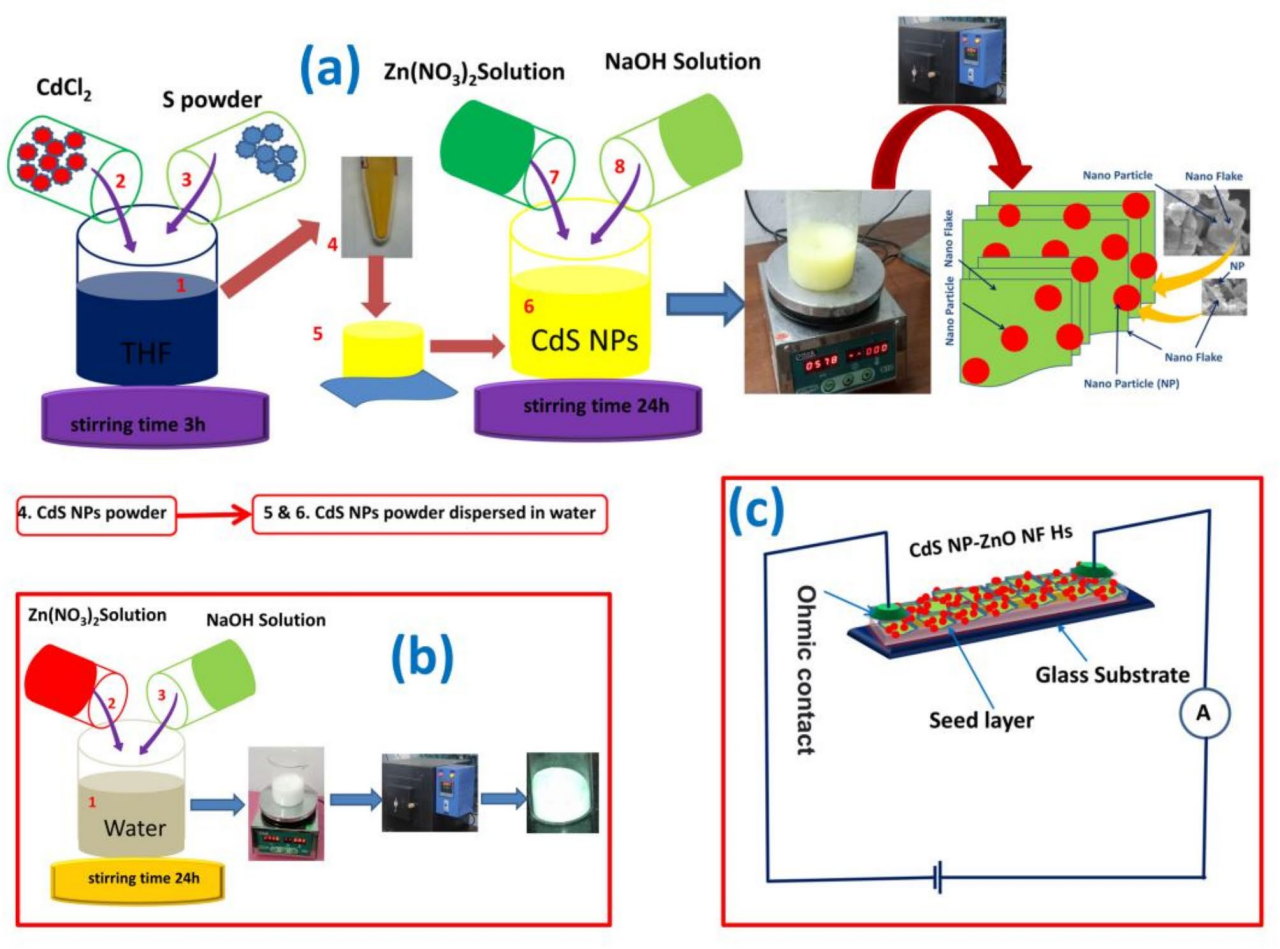
Cartoon picture of the schematic diagram for the fabrication of (a) CdS NPs and CdS NP-ZnO NF Hs, (b) ZnO NPs, (c) Schematic diagram of the device and I-V measured circuit.

Initially, CdS nanoparticles were prepared by a simple chemical precipitation process. Briefly, cadmium chloride is added in the THF solvent under magnetic stirrer with stair bars at room temperature. Then sulphur powder is added in the solution at the same condition. After 30 min magnetic stirring of the mixed solution, the NaBH_4_ was added. The stirring was continued for 3 h at the same temperature in the same physical environment. The NaBH_4_ act as a reducing agent, which reduces Cd from CdCl_2_ and then Cd combined with S in the THF formed CdS. Also, the THF solution acts as a capping agent. The final precipitation was collected after several times washing followed by centrifuge. The collected yellow color precipitated was dried at room temperature (Fig. 1(a)). The final yellow CdS powder (N1) was used for further characterization and fabrication of heterostructure. The CdS-ZnO heterostructure (N3) was fabricated by cost effective chemical precipitation techniques. Briefly, fabricated CdS NPs powder (0.1 gm) was dissolved in 50 mL water and magnetically stirred for 1 h, and then zinc nitrate solution (0.5 M) is added in the solution under constant stirring. After 30 min, NaOH solution (0.2 M) is added in the solution. The color of the solution becomes mixture of yellow-white (Fig. 1 (a)). The mixed solution is magnetically stirrer for 24 h. The final precipitation is collected followed by centrifuge and washing several times with water and alcohol. The final powder was annealed at 70 °C for 2 h to enhance the crystallites of the material. Without using CdS NPs powder, the naocrystalline ZnO (N2) was fabricated using same process with the use of same amount of precursors followed by annealing at 400 °C for 4 h of the final powder (Fig. 1(b)).

### Instrumentations for different characterization

Different instruments are used for characterization of the fabricated nanocrystalline semiconductors. XRD of the powder samples have been recorded with the use of Rigaku X-ray diffractometer with angular rages 20° to 80° having Cu-K_α_ radiation of wavelength 1.54 A^°^. Field emission scanning electron microscopy (FESEM) images were taken with the use of Zeiss FESEM microscope (operating voltage 5 kV). The optical spectrum in the UV-Vis region of the fabricated samples have been taken after dispersed in water with the use of Shimadzu-Pharmaspec-1700-UV-Vis spectrometer (wavelength ranges 200 nm to 800 nm). The current (I)-voltage (V) characterization of the samples deposited on glass substrate with ohmic contact were measured with the use of ‘Agilent 4156 C Precision Semiconductor Parameter Analyser along with Agilent 41501B SMU’ (Fig. 1(c)). The photocatalytic activity were investigated with the monitoring UV-Vis spectroscopy recorded in Shimadzu-Pharmaspec-1700 UV-Vis spectrometer (wavelength ranges 200 nm to 800 nm) of the MB dye (in water medium) in presence of visible light irradiation (white light bulb as a source).

## Results and discussion

### FESEM images

**Fig. 2:**
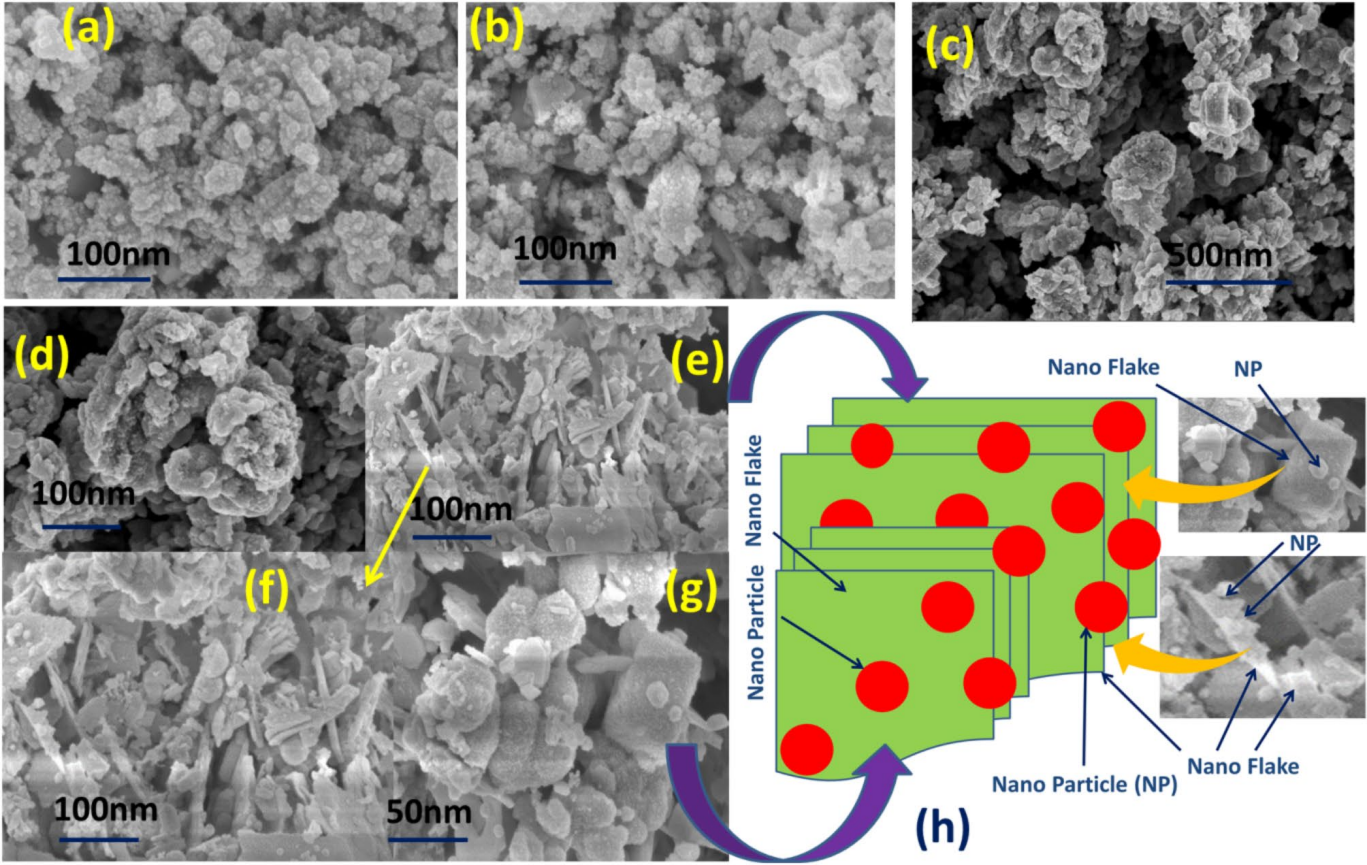
FESEM images of the: **(a)** & **(b)** CdS NPs (N1), **(c)** & **(d)** ZnO NPs (N2), **(e)** & **(f)** & **(g)** CdS NPs-ZnO NFs heterostructure (N3), **(h)** schematic diagram of the array of the heterostructure with enlarge view (zoom of (e) & (g)) of the FESEM images of CdS NPs-ZnO NFs heterostructure.

FESEM images for N1, N2, and N3 are shown in Fig. 2. The nanoparticle structure is found from the images of N1 (Fig. 2(a) & (b)). The microscopic image of N2 shows formation of small plate like structure (Nanoplates (NPs)) (See Fig. 2(c) & (d)). The heterostructure shows particles are attached with nanoflakes (NF). An array of particle-flake heterostructure is formed (Fig. 2(e) & (f), (g)). Hence, particle-flake array type heterostructure is clearly observed from the FESEM images and the schematic diagram of the array of the Hs is shown in Fig. 2(h). The particle structure is the CdS NPs, which previously fabricated and used with in the matrix of in-situ growth of ZnO. The nano flake structure comes from the ZnO, that grown during Hs fabrication.

### XRD study of the CdS NPs, ZnO NPs and CdS NP-ZnO NF heterostructures

**Fig. 3:**
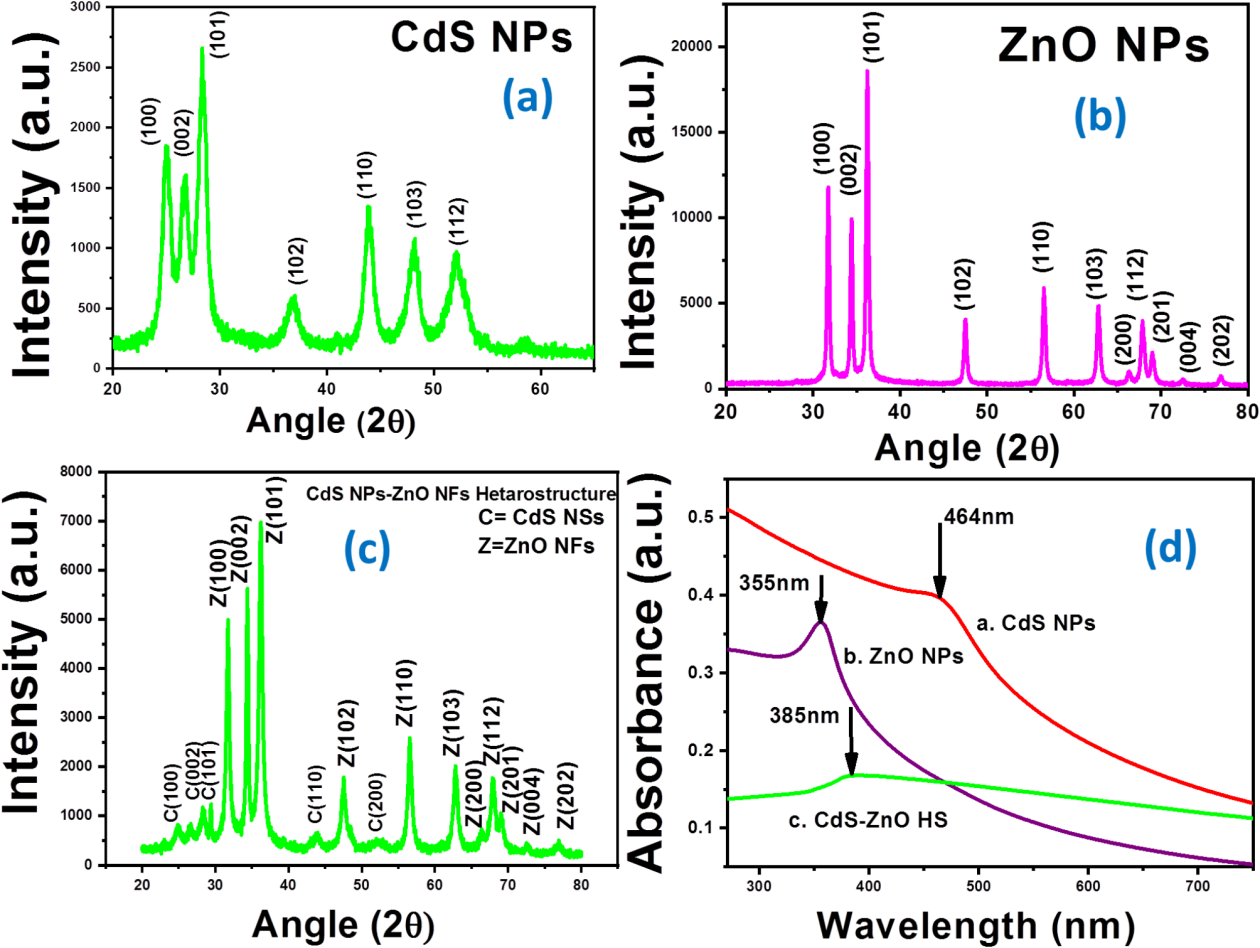
XRD pattern of the: **(a)** CdS NPs (N1), **(b)** ZnO NPs (N2), **(c)** CdS NPs-ZnO NFs heterostructure (N3); **(d)** UV-Vis spectrum of the CdS NPs, ZnO NPs, and CdS NPs-ZnO NFs Hs.

The crystalline phase of all the fabricated samples (N1, N2, N3) were studied by XRD (Fig. 3).The CdS NPs (N1) shows presence of (100), (002), (101), (102),(110), (103), (112) diffraction peaks (Fig. 3(a)), which indicated a pure hexagonal wurtzite phase of the CdS NPs^25^ .The presence of the (100), (002), (101), (102), (110), (103), (200), (112), (201), (004), (202) diffraction peaks of the N2 (Fig. 3(b)) indicated hexagonal wurtzite crystal phase of ZnO^26^. The wurtzite ZnO and hexagonal wurtzite CdS peaks are the only ones visible in the N3 XRD patterns (shown in Fig. 3(c)). The hexagonal wurtzite CdS is represented by the following peaks: (100), (002), (101), (110), (200) and that from wurtzite ZnO (100), (002), (101), (102), (110), (103), (200), (112), (201), (004), (202) indicating that the fabricated material was heterostructures comprising of hexagonal wurtzite ZnO and hexagonal wurtzite CdS i.e. lattice phase matching has been achieved^23^. In order to fabricate a heterostructure, lattice phase matching is crucial^27^.

### UV-VIS spectroscopy and opto-structural parameters

The UV-VIS spectrum of the CdS NPs (N1), ZnO NPs (N2), CdS NP-ZnO NF Hs (N3) are shown in Fig. 3(d). The peak corresponds to exciton formation in the semiconductor CdS NPs, ZnO NPs and CdS NP-ZnO NF Hs arises at 464 nm, 355 nm and 380 nm, respectively^28,29^. The room temperature exciton formation in ZnO NPs is more prominent compare with N1 and N3, whereas it’s less in CdS NP-ZnO NF HSs. The direct band gap energy (E_g_) of the N1, N2, N3 are calculated from Tauc’s equation (See supplementary S1)^30^:

1 $$\:{\left(\alpha\:h\nu\:\right)}^{2}=Constant\:(h\nu\:-{E}_{g})\:$$

**Fig. 4:**
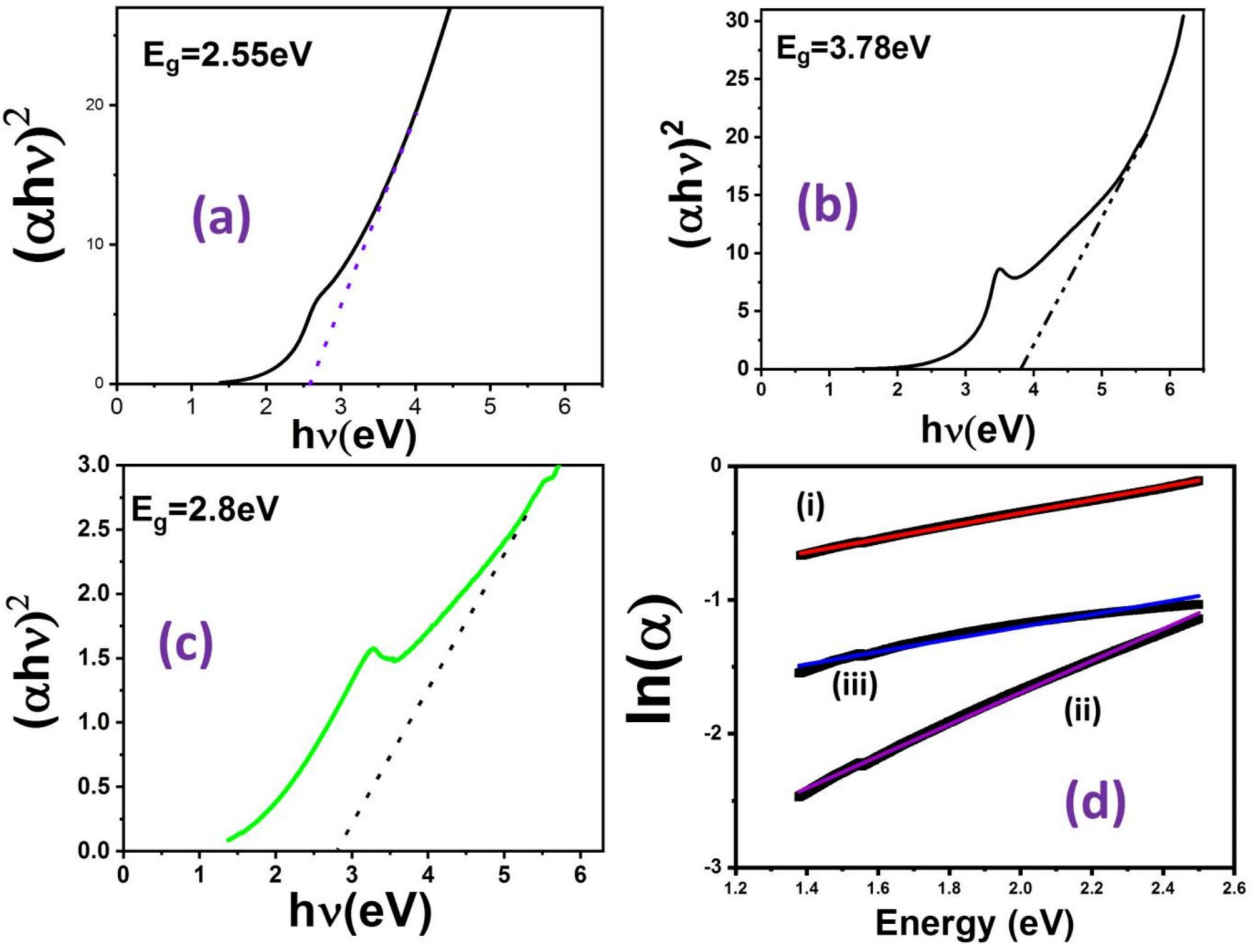
Variation of (αhυ)^2^ vs. hυ for: (a) CdS NPs (N1), (b) ZnO NPs, (c) CdS NP-ZnO NF heterostructures (N3); (d) Variation of ln(α) vs. hυ and its linear fitted straight line for the (i) CdS NPs, (ii) ZnO NPs, and (iii) CdS NP-ZnO NF heterostructures.

After plotting (αhν)^2^ vs. hν (see Fig. 4 (a), (b), (c)), the band gap (E_g_) found to be 2.55 eV, 3.78 eV and 2.8 eV, for N1, N2, N3, respectively. Tuning of the excitonic peaks from higher UV region (355 nm) of ZnO NPs to visible region (464 nm) of CdS NP-ZnO NF Hs and tuning of the band gap energy from 3.78 eV to 2.8 eV have been achieved for effective generated photo activation (visible light ) carriers for application of photo-catalytic effect. Hence, the less effective visible light response of the high band gap (3.78 eV) of the ZnO NPs is converted into a more effective visible light response band gap in the CdS NP-ZnO NFs heterostructure. The disorderedness in nanostructure is qualitatively measured with the help of Urbach energy (E_U_) (See S2) as per the below relation^31^:

2 $$\:\alpha\:={\alpha\:}_{0}{exp}^{\frac{h\nu\:}{{E}_{U}}}$$

The calculated value of the E_U_ from the straight-line fitted results of the variation of ln(α) vs. hν (Fig. 4(d)) are found to be 1.75 eV, 1 eV, 2.35 eV for CdS NPs, ZnO NPs and CdS NP-ZnO NF Hs, respectively. The result showed ZnO NPs are more ordered and CdS NP-ZnO NF Hs are less ordered within the nanoscale. The less ordered nanostructure in its small scale produces effectively high surface free energy, defects and specific surface properties, which leads more response during photocatalytic effect. Sharma et al. showed that nanomaterial with higher Urbach energy have higher degradation properties^9^.

**Fig. 5:**
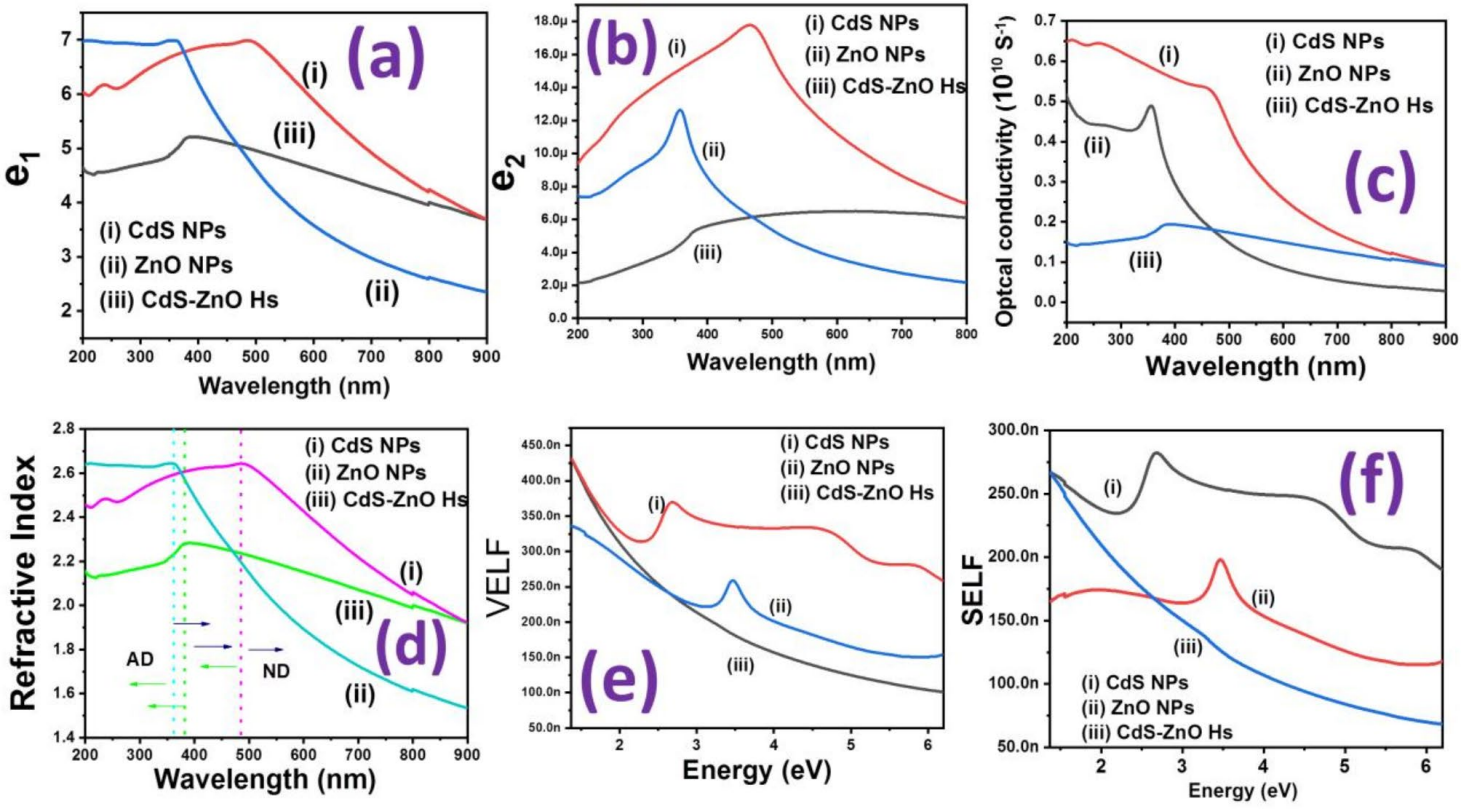
Variation of **(a)** real optical dielectric constant (e_1_), **(b)** imaginary optical dielectric constant (e_2_), **(c)** Optical conductivity (σ_Opt_), and **(d)** Refractive index (n) as a function of wavelength (λ) for (i) CdS NPs, (ii) ZnO NPs, and (iii) CdS NP-ZnO NF heterostructures; Variation of the energy loss functions: **(e)** surface energy loss function (SELF), **(f)** volume energy loss function (VELF) with photon energy (eV) for (i) CdS NPs, (ii) ZnO NPs, and (iii) CdS NP-ZnO NF heterostructures.

The modelling of the optical and electrical properties of the nanocrystalline semiconductor is expressed by two critical parameters^32^: (i) real optical dielectric constant (e_1_), (ii) imaginary optical dielectric constant (e_2_) (see S3). The variation of e_1_ and e_2_ as a function of wavelength for N1, N2, N3 are shown in Fig. 5(a) & (b), respectively. The parameters optical conductivity (σ_Opt_) and refractive index (n) are also analysed (S3)^32^. The resultant variation of optical conductivity (σ_Opt_) and refractive index with wavelength are shown in Fig. 5(c), (d) for N1, N2, N3. Below 470 nm wavelength σ_Opt, CdS NPs_ > σ_Opt, ZnO NPs_ > σ_Opt_, _Hs_ and above 470 nm wavelength σ_Opt, CdS NPs_ > σ_Opt_, _Hs_ > σ_Opt, ZnO NPs_. The enhanced absorbance of the nanostructures is the cause of the higher optical conductivity at high photon energies (hν) in all samples. Its value for CdS NPs ranges from 0.09 × 10^9^ S^− 1^ to 0.65 × 10^9^ S^− 1^, ZnO NPs ranges from 0.03 × 10^9^ S^− 1^ to 0.5 × 10^9^ S^− 1^, CdS NP-ZnO NF Hs ranges from 0.09 × 10^9^ S^− 1^ to 0.15 × 10^9^ S^− 1^. The refractive index for CdS NPs ranges from 1.9 to 2.45, ZnO NPs ranges from 1.5 to 2.65, CdS NP-ZnO NF Hs ranges from 1.9 to 2.15.The normal dispersion regions (dn/dλ=-ve) (ND) and anomalous dispersion regions (dn/dλ=+ve) (AD) are separated by dotted line and marked with arrow sign (Fig. 5(d)). Two important energy loss (functions volume energy loss function (VELF) and surface energy loss function (SELF)) (see S4) are analysed after plotting their variation with electromagnetic energy (see Fig. 5 (e) & (f)). Both figures show that the rate of change of VELF in relation to energy is faster than the similar changes of SELF. The resultant variation of optical conductivity and VELF, SELF shows that CdS NP-ZnO NF Hs is better for optical responsive material compare with pure ZnO NPs and CdS NPs.

### Time-correlated single photon counting (TCSPC) spectrum and ultrafast life time of the heterostructure

**Fig. 6:**
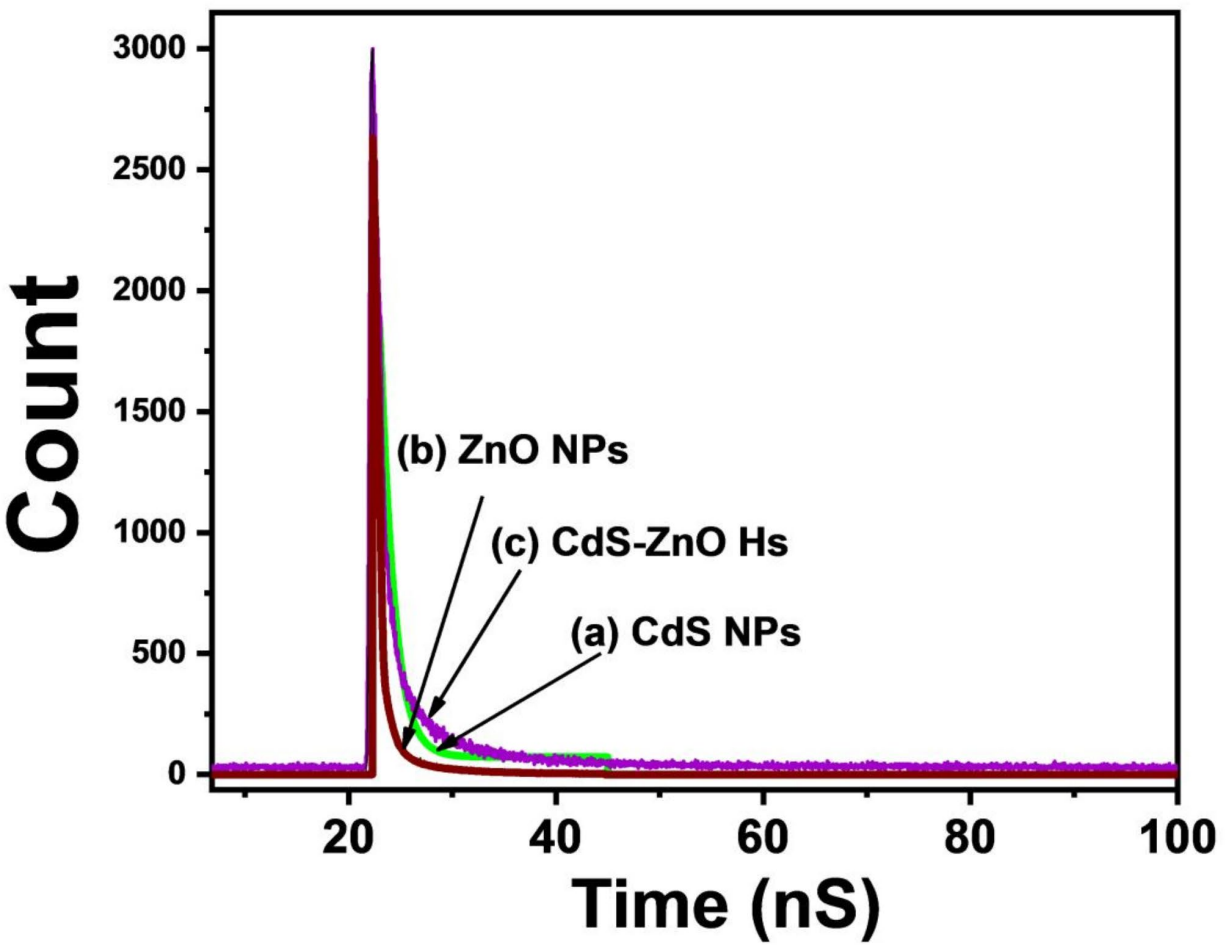
The kinetic of fluorescence decay (TCSPC) of (a)CdS NPs, (b) ZnO NPs, (d) CdS NP-ZnO NF heterostructure.

The time-correlated single photon counting spectrum (TCSPC) of the CdS NPs, ZnO NPs and CdS NP-ZnO NF Hs are shown in Fig. 6. The average life time obtain from the decay graphs are 17.4 ns, 4.41 ns, and 59 ns for CdS NPs, ZnO NPs and CdS NP-ZnO NF Hs, respectively. The life time of the charge carriers enhances in the Hs compare with pure CdS NPs and ZnO NPs because of the formation of the large number of free charges in the Hs^19,33^.

### Current (I)–voltage (V) study

**Fig. 7:**
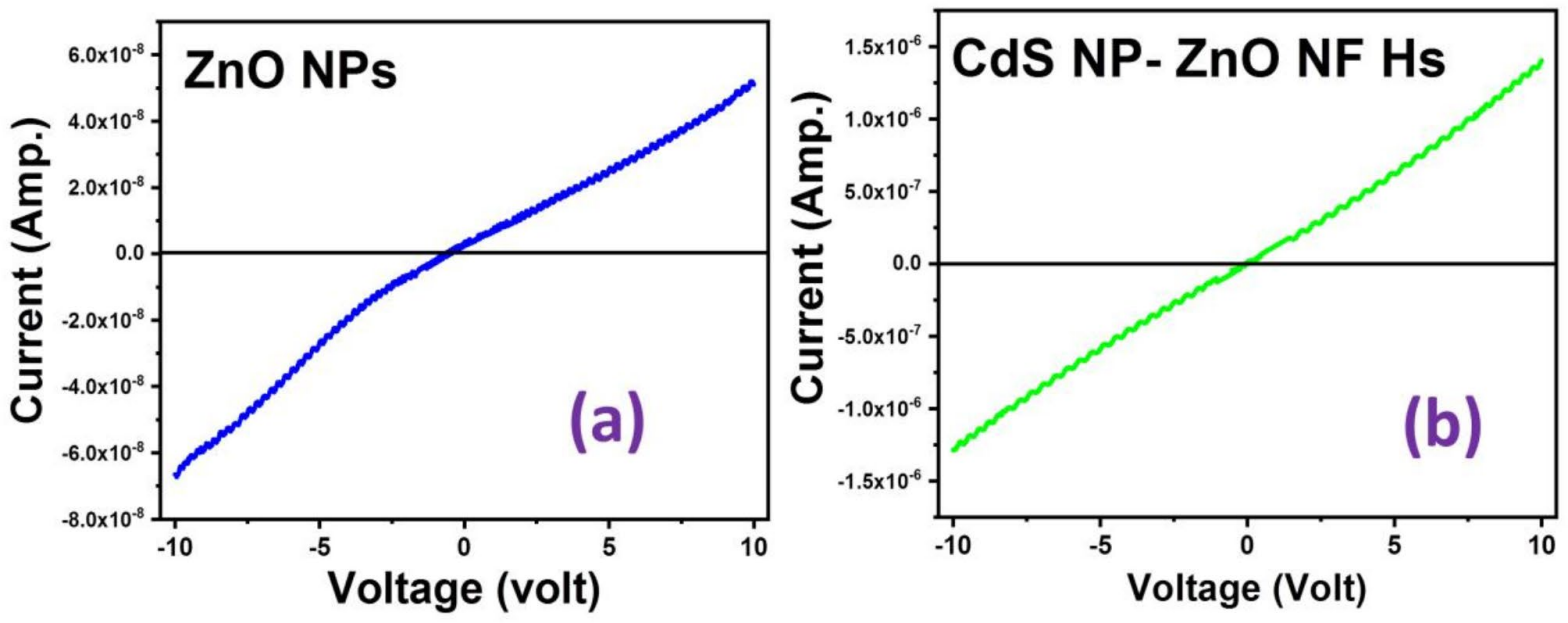
Room temperature current (I) - voltage (V) graph of the ZnO NPs and CdS NP-ZnO NF heterostructure.

The current (I) - voltage (V) characteristics of the ZnO NPs and CdS NP-ZnO NF Hs have been investigated within the voltage ranges − 10 V to + 10 V (Fig. 7). The current at the voltage + 10 V for the ZnO NPs and CdS NPs-ZnO NFs Hs are 5.2 × 10^− 8^ A and 1.5 × 10^− 6^ A, respectively. The interfacial charge separation in the nanoscale heterostructureand reduction of the band gap in the heterostructure compare with pure ZnO NPs leads to enhance/ increase of the room temperature current^19^. This enhancement comes from the availability of the more free charges in the Hs due to interfacial charge transfer and lowering band gap compare with pure ZnO NPs.

### Photocatalytic activity: photo-degradation of methylene blue (MB)

#### Photodegradation and reaction kinematics

The degradation of the MB dye under visible light irradiation of 28 min duration is studied separately in the presence of CdS NPs (N1), ZnO NPs (N2), and CdS NP-ZnO NF Hs (N3). At first, 1 mg MB dye dissolved in 100 mL normal water. Then the required amount (10 mg or 5 mg) of particular nanomaterials (N1/N2/N3) was directly added to the MB solution just before the light irradiation. For each dose of different nanomaterials (N1/N2/N3), separate MB solutions (1 mg in 100 mL) were prepared, and different nanomaterials (N1/N2, N3) with required doses were directly added to separate MB solutions just before the photocatalytic degradation study of the particular solution. During light illumination, the particular solution was kept under constant magnetic stirring. The degradation effect is studied by monitoring the UV-VIS absorption spectrum of a particular MB solution in the presence of particular nanomaterial with different light illumination time. After a complete study of the effect of a particular nanomaterial with a particular dose on MB solution, the same process was repeated for other doses and other nanomaterials one by one.

**Fig. 8:**
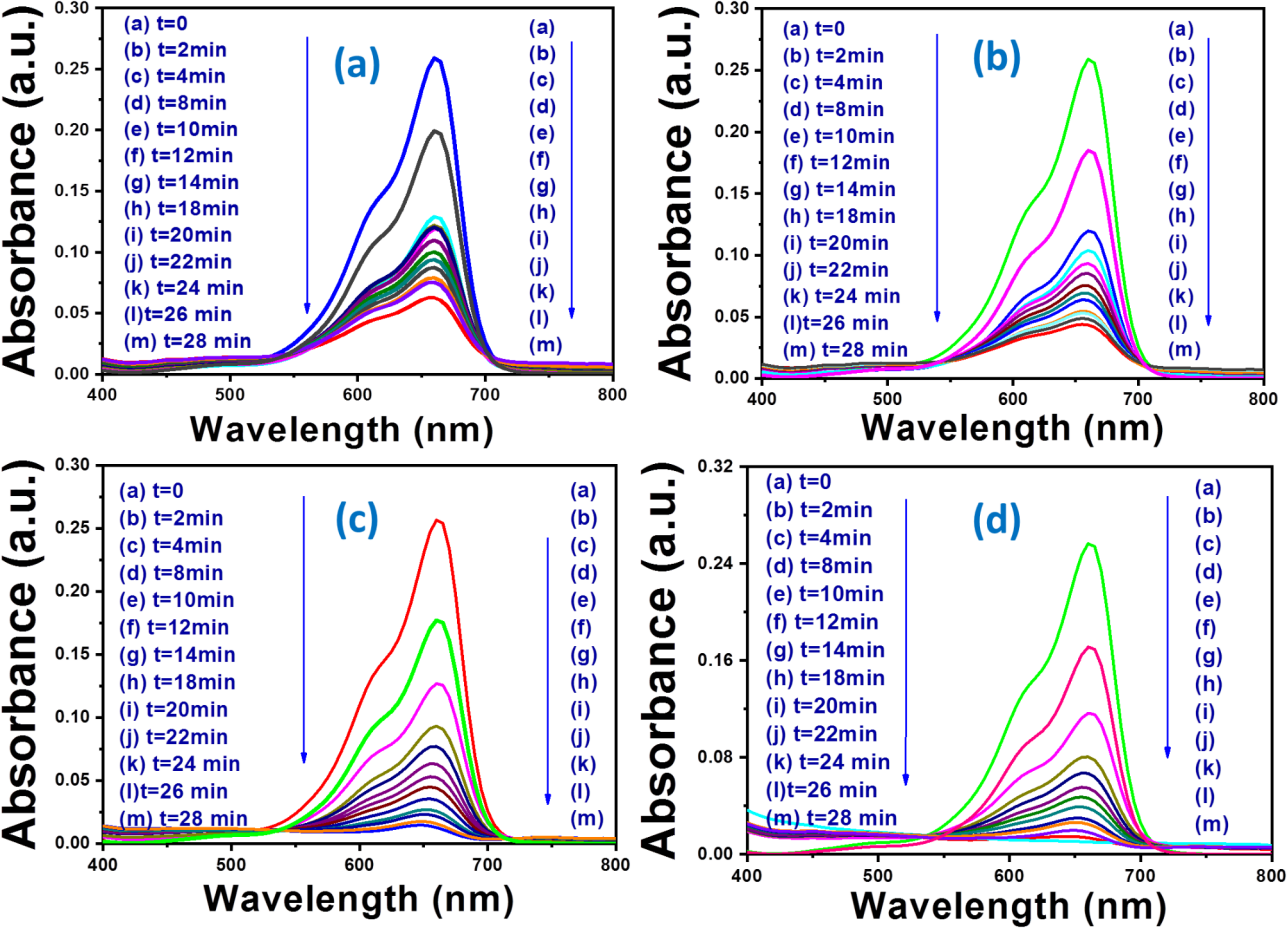
: Photodegradation of MB under visible light in presence **(a)** ZnO NPs with dose 10 mg, **(b)** CdS NPs with dose 10 mg, **(c)** CdS NPs-ZnO NFs with dose 5 mg, **(d)** CdS NPs-ZnO NFs Hs with dose 10 mg.

**Fig. 9:**
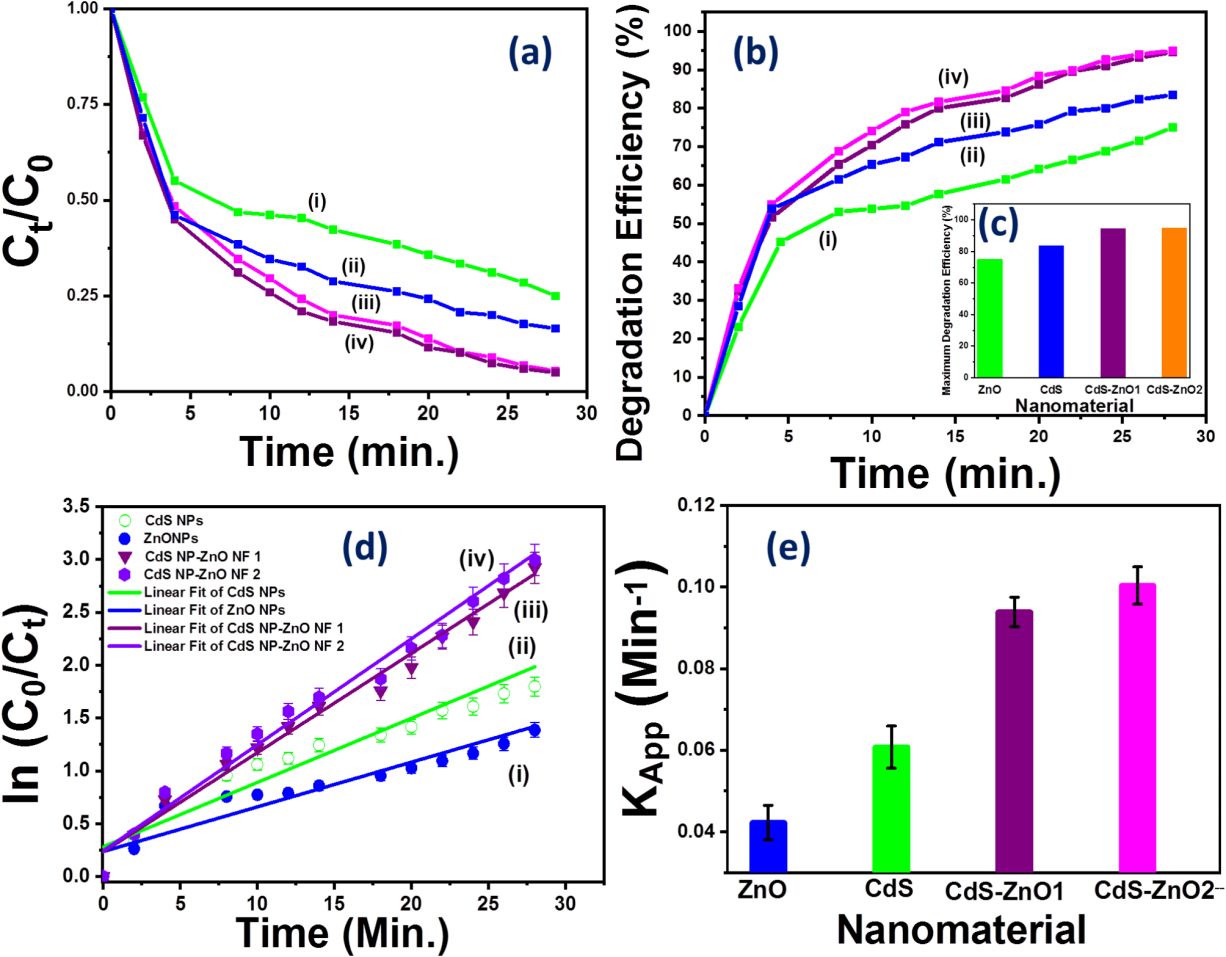
**(a)** Variation of relative concentration vs. time for (i) ZnO NPs, (ii) CdS NPs, (iii) CdS NP-ZnO NF Hs dose 5 mg, (iv) CdS NP-ZnO NF dose 10 mg; **(b)** Variation of the degradation efficiency (%) vs. time for (i) ZnO NPs, (ii) CdS NPs, (iii) CdS NP-ZnO NF Hs dose 5 mg, 10 mg (iv) CdS NP-ZnO NF Hs dose 10 mg ; **(c)** Histogram showing maximum degradation efficiency (%) for different nanomaterial ; **(d)** Variation of ln(C_0_/C_t_) vs. time (t) for for (i) CdS NPs, (ii) ZnO NPs, (iii) CdS NPs-ZnO NFs dose 5 mg, (iv) CdS NPs-ZnO NFs dose 10 mg ; **(e)** Histogram showing variation of apparent rate constant (K_app_) for different nanomaterial.

The photodegradation of MB under visible light in the presence of ZnO NPs, CdS NPs, and CdS NPs-ZnO NFs is shown in Fig. 8. The absorbance MB and hence the concentration of the pure MB reduces in presence of light irradiation time with the use of nanostructure semiconductors (N1, N2, N3). The degradation of the MB dye is clearly observed (absorbance and concentration of MB reduces compare with pure MB) in presence of each nanocrystals and degradation is different for the use of different nanomaterials with in 28 min irradiation time (Fig. 8). The changes in the relative concentration i.e. ratio of concentration of the MB at time t (C_t_) to concentration of the MB at the time t = 0 min (C_0_) in presence of semiconductor nanostructure with time of the visible light irradiation for the use of the ZnO NPs, CdS NPs and CdS NP-ZnO NF Hs are shown in Fig. 9(a).

**Table 1:** Different optical, structural and photocatalytic parameters.

Sample	E_g_ (eV)	E_U_ (eV)	Crystal Phase	Average life time (nS) from TCSPC	Maximum current at + 10 V (Ampere)	K_app_ (min^− 1^)	Maximum Degradation efficiency within 28 min irradiation of Light
CdS NPs	2.55	1.75	Pure hexagonal wurtzite phase	17.4	-	0.061 ± 0.0052 (5 mg)	83.5%
ZnO NPs	3.78	1	Pure hexagonal wurtzite phase	4.41	5.25 × 10^− 8^	0.042 ± 0.0042 (5 mg)	75%
CdS NP-ZnO NFHs	2.8	2.35	Mixture of hexagonal wurtziteCdS and ZnO	59	1.4 × 10^− 6^	0.094 ± 0.0036 (5 mg)	94.6%
						0.1 ± 0.0046 (10 mg)	95%

The photocatalytic degradation efficiency (ɳ) was calculated with the use of the below relation^34,35^:

3 $$\:\eta\:=\left(\frac{{A}_{0}-{A}_{t}}{{A}_{0}}\right)\times\:100\:\%$$

Where, ‘A_0_’ is the absorbance of free MB (initial absorbance) and ‘A_t_’ is the absorbance of the MB in presence of nanomaterial under different irradiation time (t). The variation of percentage degradation efficiency (ɳ) as a function of visible light irradiation time for nanomaterial of ZnO NPs and CdS NPs with doses 10 mg, CdS NP-ZnO NF HSs with doses 5 mg and 10 mg are shown in Fig. 9(b). The maximum degradation efficiency (ɳ) in presence of 5 mg and 10 mg doses of CdS NP-ZnO NPHs is found to be almost same with the value 94.6% and 95%, respectively. Whereas for the use of 10 mg doses of ZnO NPs and CdS NPs, the ‘ɳ’ value is found to be 75% and 83.5%, respectively. Comparatively higher value of the ‘ɳ’ have been achieved for CdS NP-ZnO NF Hs (95%) compare with same dose (10 mg) and same irradiation time (28 min) of visible light for ZnO NPs (75%) and CdS NPs (83.5%). The maximum degradation efficiency at the end product is shown by a histogram in the Fig. 9(c). This highly efficient activity of the Hs was induced by enhanced charge separation under light irradiation in the nanocrystal heterostructure semiconductors as shown by the band diagram in Fig. 11. The precise photodegradation efficiency (ɳ) values at the end of the final product following a 28 min exposure to visible light (end of the observation time) are reported in Table 1. The following equation, which takes into account the first order reaction, explains the reaction mechanism during the photocatalytic process^34,36,37^:

4 $$\:\text{ln}\left(\frac{{C}_{0}}{{C}_{t}}\right)=\:{K}_{app}\:t$$

Where, K_app_ = apparent rate constant. The variation of ln (C_0_/C_t_) vs. time (t) for N1, N2, N3 are shown in Fig. 9(d). The linear fitted of the variation of ln (C_0_/C_t_) with time gives the value of K_app_. The K_app_ value for the use of ZnO NPs, CdS NPs, and CdS NP-ZnO NF Hs are found to be 0.042 ± 0.0042 (min)^−1^, 0.061 ± 0.0052 (min)^−1^ and 0.1 ± 0.0046 (min)^−1^, respectively. The calculated value of the apparent rate constant (K_app_) for the use of N1, N2, N3 are listed in Table 1. The apparent rate constant (K_app_) for the use of different nanomaterials (ZnO NPs, CdS NPs, and CdS NP-ZnO NF Hs) as photocatalyst is shown by a histogram in the Fig. 9(e). The degradation efficiency (ɳ) as well as K_app_ increases from ZnO NPs to CdS NP-ZnO NF Hs (N3 > N1 > N2). Hence, the Hs accelerated the photocatalytic process. This is may be due to the extra surface charge appears at the interfaces of the heterostructure (Fig. 11). Compare with nanostructure CdS and ZnO, the band of CdS NPs (486 nm) lies in the visible region and that of ZnO NP (328 nm) lies at the interface of lower visible and upper UV region whereas the band of CdS NP-ZnO Hs (443 nm) goes inside visible region. Also, higher Urbach energy and greater ultrafast life time of the CdS NP-ZnO Hs leads to enhanced photocatalytic response.

#### Reusability and stability

**Fig. 10:**
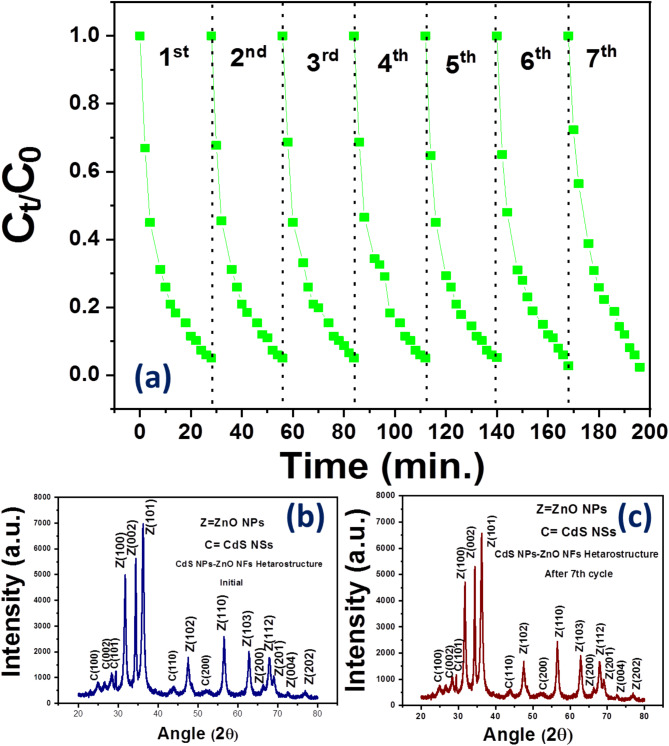
**(a)** Cyclic degradation experiment (showing relative concentration vs. time for seven consecutive cycles) for CdS NP-ZnO NF Hs with dose 10 mg ; (b) XRD of the catalyst CdS NP-ZnO NFs before 1st cycle ; (c) XRD pattern of the same catalyst i.e. CdS NP-ZnO NFs after 7th cycle.

The stability of the photocatalytic activity of CdS NP-ZnO NP Hs with doses 10 mg were studied by cyclic degradation experiment with examine seven cycles (variation of relative concentration with time) shown in Fig. 10 (a). After 7th cycle the degradation rate (efficiency 94.75%) changes slightly compare to 1st cycle (95%). This very small change in the degradation rate after seventh cycle test arises due to very small mass loss of the catalyst. Also, XRD of the catalyst CdS NP-ZnO NFs was taken after 7th cycle (see Fig. 10(c)), which shows no changes in diffraction peak position of the catalyst. The intensity of XRD pattern of the CdS NP-ZnO NFs after 7th cycle (Fig. 10 (c)) shows a very small decrement of the intensity compare with its initial state (before 1st cycle) (Fig. 10(b))^38^. Hence the HSs were very stable photocatalyst and showed better reusable properties.

#### Mechanism of photocatalytic activity for the heterostructure photocatalyst

The particular photocatalyst area of the heterostructure and the coupling effects of ZnO NFs and CdS NPs are two potential explanations for the augmentation of the photocatalytic activity of the heterostructure photocatalyst.

**Fig. 11:**
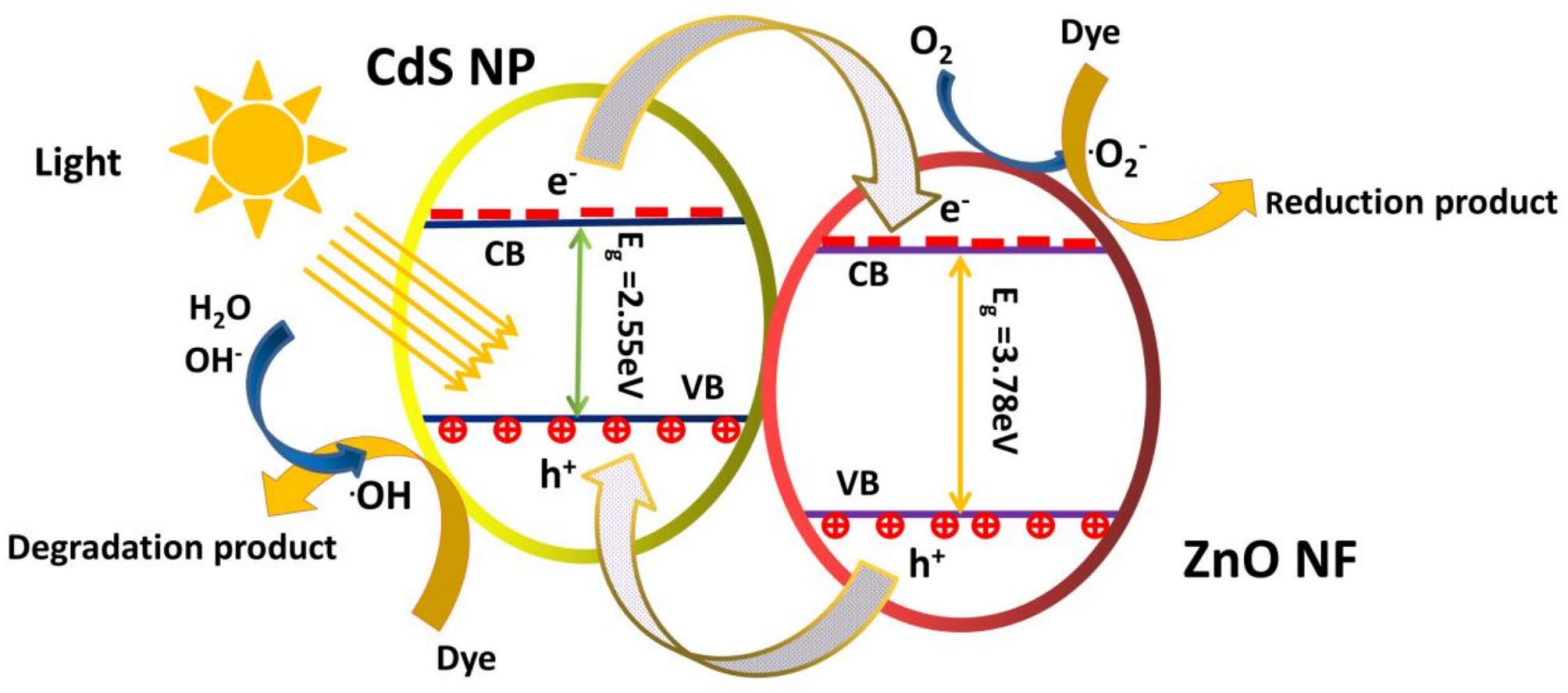
Schematic diagram of the Photocatalytic mechanism of type II heterojunction with band structure.

The enhanced photocatalytic activity of the CdS NPs-ZnO NFs Hs can analysed as per the band diagram of the Fig. 11. The type-II structure of the band positions of CdS NPs and ZnO NFs is depicted in the schematic diagram (Fig. 11). In each semiconductor material (ZnO NF and CdS NP, for example), photo-generated electron–hole pairs can be effectively separated and their recombination reduced by this type-II band alignment^39,40^. The light activated carrier produced (under visible light irradiation condition) in the CdS NPs side (conduction band of CdS NPs) on the CdS NP-ZnO NF HSs were accelerated to the conduction band of the ZnO NFs. This movement of light activated electrons from the conduction of CdS NP to the conduction band of ZnO NF with in the hetero-structure involved very quickly in the photocatalytic process.

The hydroxyl radical OH°, a potent oxidising agent that breaks down organic dyes, is produced when photogenerated electrons go to the conduction band of ZnO NFs and convert molecular oxygen O_2_ to the superoxide radical anion O_2_^**°**−^. The following is a description of the suggested mechanism for the photocatalytic degradation of organic dye by CdS NP-ZnO NF heterostructures^22,23,41^:

$${\text{CdS NP}} - {\text{ZnO NF Hs+ h}}\upsilon \xrightarrow{{Irradiation}}\,~{\text{CdS NP }}\left( {{{\text{h}}^+}} \right) - {\text{ZnO NF }}({{\text{e}}^ - })$$

$${\text{ZnO NF}}({{\text{e}}^ - })\,+\,{{\text{O}}_{\text{2}}}\xrightarrow{{irradiation}}{\text{O}}_{2}^{{. - }}$$

$${\text{O}}_{2}^{{. - }}+{\text{ }}{{\text{H}}_{\text{2}}}{\text{O}}\xrightarrow{{irradiation}}~{\text{H}}~{\text{O}}_{2}^{.}+{\text{ O}}{{\text{H}}^ - }$$

$$~{\text{HO}}_{2}^{.}+{\text{water}}~\xrightarrow{{irradiation}}{{\text{H}}_{\text{2}}}{{\text{O}}_{\text{2}}}+{\text{O}}{{\text{H}}^.}$$

$${{\text{H}}_{\text{2}}}{{\text{O}}_{\text{2}}}\xrightarrow{{Irradiation}}2{\text{O}}{{\text{H}}^.}$$

$$~{\text{O}}{{\text{H}}^.}+{\text{MB dye}}\xrightarrow{{Irradiation}}~{\text{C}}{{\text{O}}_{\text{2}}}\,+\,{{\text{H}}_{\text{2}}}{\text{O}}$$

## Conclusion

In conclusion, a simple two-step solution method (chemical precipitation method) has been successfully implemented to prepare CdS NP-ZnO NF heterostructure arrays. In comparison to CdS NPs and ZnO NPs alone, it was observed that the CdS NP-ZnO NF heterostructure arrays had better photocatalytic activity. I anticipate that the CdS NP-ZnO NF heterostructure arrays, with their effective charge separation process and interfacial charge transfer properties, will present a promising material and application for photocatalytic degradation of water pollution, photoelectrodes, applications in photovolatics, and solar-energy conversion nanomaterials. Lattice structure of both nanostructures of CdS and ZnO showed hexagonal phase, which makes better coupled system for type-II hetarostructure. As-prepared heterostructures comprising of hexagonal wurtzite ZnO and hexagonal CdS i.e. lattice phase matching has been achieved. The incorporation of CdS NPs within ZnO NFs improved the optical light response and efficient visible region photocatalytic properties of the Hs with enhancing charge carriers and reducing band gap of the pure ZnO NPs from 3.78 eV to Hs ≈ 2.8 eV. The heterostructure showed enhanced carrier life time, electrical current compare with ZnO NP and CdS NP. This work offers a novel concept and path forward for the study of heterostructure carriers. It also offers a workable way to degrade water contamination using photocatalysis, and promising material for the fabrication of optoelectronic devices.

## Electronic supplementary material

Below is the link to the electronic supplementary material.

Supplementary Material 1

## Data Availability

‘’All data that support the findings of this study are included within the article’’.
