# Supplementary material for: Type-II heterostructure of semiconducting CdS nanoparticle-ZnO nanoflake arrays for visible light dependent enhanced photocatalytic activity
Source: Sci Rep. 2025 May 2;15:15364. doi: 10.1038/s41598-025-88141-x (PMC12048577; doi:10.1038/s41598-025-88141-x)
Supplement: Supplementary file 1 — Supplementary Material 1 [file 41598_2025_88141_MOESM1_ESM.docx]

## **Type-II heterostructure of semiconducting CdS nanoparticle-ZnO nanoflake arrays for visible light dependent enhanced photocatalytic activity**

**Amit Kumar Bhunia^1*^**

^1^Department of Physics, Government General Degree College Gopiballavpur-II, Jhargram- 721517, India

*Email: [amitphysics87@gmail.com](mailto:amitphysics87@gmail.com)

**S1: Tauc’s equation**:

Relation among optical absorption coefficient (α) and band gap energy (E_g_) is given by the Tauc’s equation [1]:

${(\alpha h\nu)}^{2}=Constant (h\nu-E_{g})$……………………………………………….(1)

Where ν is the frequency of the electromagnetic wave.

**S2: Urbach energy**:

Urbach energy (E_u_) is calculated from the following relation [2]:

$\alpha=\alpha_{0}{exp}^{\frac{h\nu}{E_{U}}}$ ………………………………………………………….(2)

Where ‘α’ is the absorption coefficient and ‘h’ is the Planks constant.

**S3: Different Optical and structural parameters:**

The two important optical dielectric constants are (i) real optical dielectric constant (e_1_), (ii) imaginary optical dielectric constant (e_2_). The value of e_1_ and e_2_ computed from the below relations [3]:

$\varepsilon_{1}=n^{2}-K^{2}$ ……………………….……………………………………………………(3)

$\varepsilon_{2}=nK$ ……………………………………………………………………………..........(4)

Where n = refractive index. The value of n is related with the extinction coefficient (K), reflectance (R) by the below relation [3]:

$n=\frac{1+R}{1-R}+\sqrt{[ \left\{ \frac{4R}{\left( 1-R \right)^{2}} \right\} -K^{2} ]}$…………..………………………………………………….(5)

Another important parameter is the conductivity (σ_Opt_) of the semiconductor with thickness (t = unit) is defined by the below relations

$\sigma_{Opt}= \frac{n\alpha c}{4\pi}$ ………..……………………………………………………………………....(6)

**S4: Energy loss functions:**

Two important energy loss functions of the semiconductor nanomaterials are (i) surface energy loss function (SELF) and (ii) volume energy loss function (VELF). The SELF and VELF are computed with the help of below relations [1]:

$SELF=\frac{\varepsilon_{2}}{\{\left( \varepsilon_{1}+1 \right)^{2}+{\varepsilon_{2}}^{2}\}}$……………………………………………………………..…………(7)

$VELF=\frac{\varepsilon_{2}}{\{{\varepsilon_{1}}^{2}+{\varepsilon_{2}}^{2}\}}$………………………………………………………………….………..(8)

**References**

**[1]** A.K. Bhunia, S. Sen, P.K. Guha, S. Saha, Negative photoconductivity: optical and structural characterization of PVP encapsulated CuO nanorods for the study of negative photoconductivity effect, The European Physical Journal Plus 138 (2023). https://doi.org/10.1140/epjp/s13360-023-04244-2.

**[2]** A.K. Bhunia, P.K. Jha, S. Saha, Exciton–tryptophan coupling pulse behaviour along with corona formation, binding analysis, and interaction study of ZnO nanorod–serum albumin protein bioconjugate, Luminescence 37 (2022) 892–906. https://doi.org/10.1002/bio.4233.

**[3]** A.K. Bhunia, S. Saha, Characterization of budding twigs of flower-type zinc oxide nanocrystals for the fabrication and study of nano-ZnO/p-Si heterojunction UV light photodiode, Journal of Materials Science: Materials in Electronics 32 (2021) 9912–9928. https://doi.org/10.1007/s10854-021-05649-4.
